# Systems Approaches to Modeling Chronic Mucosal Inflammation

**DOI:** 10.1155/2013/505864

**Published:** 2013-10-21

**Authors:** Mridul Kalita, Bing Tian, Boning Gao, Sanjeev Choudhary, Thomas G. Wood, Joseph R. Carmical, Istvan Boldogh, Sankar Mitra, John D. Minna, Allan R. Brasier

**Affiliations:** ^1^Sealy Center for Molecular Medicine, The University of Texas Medical Branch, 301 University Boulevard, Galveston, TX 77555, USA; ^2^Department of Internal Medicine, The University of Texas Medical Branch, 301 University Boulevard, Galveston, TX 77555, USA; ^3^Hamon Center for Therapeutic Oncology Research, Department of Internal Medicine Pharmacology, University of Texas Southwestern Medical Center, Dallas, TX 75390, USA; ^4^Institute for Translational Sciences, The University of Texas Medical Branch, 301 University Boulevard, Galveston, TX 77555, USA; ^5^Departments of Biochemistry and Molecular Biology, The University of Texas Medical Branch, 301 University Boulevard, Galveston, TX 77555, USA; ^6^Microbiology and Immunology, The University of Texas Medical Branch, 301 University Boulevard, Galveston, TX 77555, USA

## Abstract

The respiratory mucosa is a major coordinator of the inflammatory response in chronic airway diseases, including asthma and chronic obstructive pulmonary disease (COPD). Signals produced by the chronic inflammatory process induce epithelial mesenchymal transition (EMT) that dramatically alters the epithelial cell phenotype. The effects of EMT on epigenetic reprogramming and the activation of transcriptional networks are known, its effects on the innate inflammatory response are underexplored. We used a multiplex gene expression profiling platform to investigate the perturbations of the innate pathways induced by TGF**β** in a primary airway epithelial cell model of EMT. EMT had dramatic effects on the induction of the innate pathway and the coupling interval of the canonical and noncanonical NF-**κ**B pathways. Simulation experiments demonstrate that rapid, coordinated cap-independent translation of TRAF-1 and NF-**κ**B2 is required to reduce the noncanonical pathway coupling interval. Experiments using amantadine confirmed the prediction that TRAF-1 and NF-**κ**B2/p100 production is mediated by an IRES-dependent mechanism. These data indicate that the epigenetic changes produced by EMT induce dynamic state changes of the innate signaling pathway. Further applications of systems approaches will provide understanding of this complex phenotype through deterministic modeling and multidimensional (genomic and proteomic) profiling.

## 1. Introduction

Respiratory epithelial cells provide the principal barrier of the airways, facilitating gas exchange and mucociliary particulate clearance and are the major source of protective airway lining fluid [1, 2]. In the presence of injury, inflammation, and airway remodeling, signal transduction pathways induce global epigenetic reprogramming events to induce type II epithelial-mesenchymal transition (EMT) within a so-called epithelial mesenchymal trophic unit [3–7]. In the setting of cellular transformation, EMT is also implicated in cancer metastasis. During EMT, epithelial cells lose apicobasal polarity and decrease the expression of intercellular tight junctions (TJs), adherens junctions (AJs), and desmosomes [8, 9]. These changes lead to disruption of adhesion of the basal epithelial layer and allow cellular penetration into an extracellular matrix (ECM), promoting enhanced ECM production and fibrosis. As a result, type II EMT plays a central role in normal tissue response to injury and tissue remodeling and repair, whereas type III EMT is linked to cancer dissemination [10].

Despite the key role of EMT in normal tissue development and repair, dysregulated EMT has been proposed to be responsible for extracellular matrix (e.g., collagen) overproduction and fibrosis occurring in chronic respiratory diseases such as idiopathic pulmonary fibrosis [11, 12], asthma [13], the chronic obstructive pulmonary disease (COPD) [14], and obliterative bronchiolitis [4]. Genetic lineage studies in rodent models of airway fibrosis have shown that alveolar epithelial cells are a major source of progenitor cells for repair of the injured airway [15]. Alveolar cells undergoing EMT become dedifferentiated, capable of both self-renewal and formation of fibroblastic foci, with the latter constituting sites of active fibrogenesis. EMT-activated alveolar epithelial cells synthesize a variety of fibrogenic cytokines, including TGF*β*, TNF*α*, endothelin-1 and connective tissue growth factor (CTGF) [16], ECM components (Col 1A), and matrix metalloproteases (MMPs), all contributing to the pathological extracellular matrix remodeling. The process of dysregulated EMT has also been implicated in cancer progression. Here, EMT transition promotes growth factor independence, resistance to chemotherapeutic agents, and acquisition of stem-cell-like phenotype. These latter events are partly responsible for the maintenance of cancer and metastatic behavior. 

Mechanistic studies have shown that EMT is initiated by a wide variety of agents linked to chronic inflammation including oxidative stress (ROS) [16], growth factors (TGF, EGF, and IGF) [17], and cytokines (TNF*α*). In the airways, these factors are produced by injured epithelial cells, fibroblasts, and eosinophils [18]. The prototypical inducer of type II EMT is TGF*β*1, a cytokine master regulator that induces EMT via the canonical TGF*β*R1-Smad3-dependent signaling pathway [13]. The TGF*β*-Smad3 pathway activates downstream Wnt, notch, and NF-*κ*B signaling pathways to coordinate the complex genetic changes underlying EMT [19]. The core transcriptional regulators of the EMT program include the transcriptional regulators SNAIL (SNAI)1/2, Zebra (ZEB), Twist, and FOXC2 proteins. These factors coordinate acquisition of the mesenchymal phenotype through (1) downregulation of epithelial cadherin (E-Cad) involved in maintenance of cell polarity, (2) induction of intermediate filament proteins such as vimentin, (3) activation of small GTPases (to induce motility), and (4) expression of matrix metalloproteinases (MMPs) and collagen to induce fibrogenesis. Although EMT can be reversible, stable EMT is maintained through global epigenetic reprogramming, including the reorganization (and increase) of the transcriptionally active histone (H3) Lys (K) 36 trimethyl marks within large organized heterochromatin domains [20].

In addition to their central role in airway repair, epithelial cells also function as sentinel cells to trigger innate host response to microbial and nonmicrobial challenges/invasion [21]. These cells are activated by a plethora of processes including pathogen-associated molecular patterns (PAMPs) [22]. The presence of PAMPS triggers the innate intracellular signaling pathways, converging on the NF-*κ*B and IRF3 signaling pathways [23]. Of these, the NF-*κ*B signaling pathway is composed of two coupled pathways known as the canonical and noncanonical pathways, distinguished by the regulatory kinase and different cytoplasmic reservoir from which NF-*κ*B is activated [23–25]. In the rapidly activated canonical pathway, induced within minutes of stimulation, sequestered RelA·50 kDa NF-*κ*B1 heterodimers in the cytoplasm are liberated and enter the nucleus to activate numerous genes including proinflammatory and antiapoptotic ones [26–28]. By contrast, the slower noncanonical NF-*κ*B pathway, induced within hours of stimulation, is mediated by MAP3K14/NF-*κ*B-inducing kinase (NIK)·IKK*α* complex that processes the 100 kDa precursor into 52 kDa NF-*κ*B2 (p52), liberating the RelA·p52 heterodimer for nuclear import. We have recently shown that the slower noncanonical NF-*κ*B pathway is linked to the canonical pathway by a feed-forward module consisting of the TNF receptor (TRAF)-1 factor that complexes with, stabilizes, and activates the NIK·IKK*α* complex [24]. The noncanonical pathway induces expression of a temporally and biologically distinct group of genes [27]. 

The complex phenotype produced by EMT is beginning to be examined using an unbiased profiling coupled with computational inference [29]. Although the mechanism of inducing EMT has been extensively explored, much less is known about the effect of EMT on the network of innate inflammatory signaling pathways. By interrogating a primary human airway epithelial cell model of type II EMT using a multiplex gene expression profiling platform, we describe here how EMT perturbs the innate response. Here, we observe that the EMT phenotype dramatically affects the kinetics and patterns of the innate response. Remarkably, alterations in the NF-*κ*B pathway are mediated by transcriptional elongation mediated by enhanced phospho-Ser^2^ carboxy terminal domain (CTD) of RNA Pol II binding to innate gene promoters. Dynamic deterministic modeling shows that the accelerated expression of TRAF1 and NF-*κ*B2 in a coordinated IRES-dependent manner produces a shorter canonical-noncanonical NF-*κ*B coupling constant. These studies suggest that a global analysis of the EMT phenotype can be further examined using computational deterministic modeling, multidimensional profiling, and analysis of large-scale chromatin structure. 

## 2. Materials and Methods

### 2.1. RNA Extraction and Quantitation

Total cellular RNA was extracted using either RNAqueous phenol-free total RNA isolation kits (Life Technologies, CA) or Quick-RNA MiniPrep kits (ZYMO Research) according to the manufacture's recommendations. RNA was quantitated spectrophotometrically using a NanoDrop ND-1000 (NanoDrop Technologies, DE). The quality of the purified RNA was assessed by the visualization of 18S and 28S RNA bands using an Agilent BioAnalyzer 2100 (Agilent Technologies, CA). The resulting electropherograms were used in the calculation of the 28S/18S ratio and the RNA integrity number [30].

### 2.2. Assay Design and Validation

Real-time quantitative RT-PCR (QPCR) assays were designed from the coding sequence (CDS) of the gene of interest (NCBI), and exon-exon junctions were mapped via BLAT [31]. Whenever possible, at least one of the two PCR primers was designed to transcend an exon-intron junction in order to reduce the potential impact of genomic DNA contamination in the surveyed RNA samples. Primers were designed using Primer Express 2.0 (Applied Biosystems) with default settings (Primer *T*_*m*_ = 58°C–60°C, GC content = 30–80%, Length = 9–40 nucleotides, and Amplicon Length = 90–150 nucleotides). Primers were synthesized (IDT) and reconstituted to a final concentration of 100 uM (master stock) and a working stock of 5 uM. Specificity of each assay was confirmed via PCR under the reaction conditions listed below and analyzed by PAGE. A reverse transcriptase minus control was included to determine the existence of signal contribution from genomic DNA. Assays were formatted using a 96-well platform.

### 2.3. Real-Time QPCR Analysis of Gene Expression

Reverse transcription was performed on 1 ug of total RNA with random primers, utilizing TaqMan reverse transcription reagents (Applied Biosystems) under conditions recommended by the manufacturer. Although the mass of input RNA should not be utilized for normalization purposes, the amounts of input RNA to be assayed were equivalent. The reverse transcription product was used as a template for the subsequent PCR reaction, consisting of SYBR Green PCR Master Mix, template cDNA, and assay primers (Table 1) in a total reaction volume of 25 uL. Thermal cycling (50°C, 2 min; 95°C, 10 min; and 40 cycles at 95°C, 15 S; 60°C, 1 min) was performed using an ABI prism 7500 sequence detection system (Life Technologies, CA). Threshold cycle numbers (*C*_*t*_) were defined as fluorescence values, generated by SYBR Green binding to double stranded PCR products, exceeding baseline. Relative transcript levels were quantified as a comparison of measured *C*_*t*_ values for each reaction, normalized using a reference assay for human polymerase beta (NM_002690; Fwd: 5′ACAATCAATGAGTACACCATCCGT3′; Rev: 5′TCCTGCAACTCCAGTGACTCC3′) and compared to those of a “control sample” (SAEC TGF*β* minus) [32]. 

### 2.4. Cell Culture and EMT Transformation

The primary (nonimmortalized) human small airway epithelial cell (HSAEC) was purchased from Lonza. An immortalized cell line was established by infecting primary HSAECs with human telomerase (hTERT) and cyclin dependent kinase (CDK) 4 expressing retrovirus constructs and selecting under 250 ng/mL puromycin and 30 *μ*g/mL G418 as described in [33]. The immortalized HSAECs were grown in small airway epithelial cell growth medium (SAGM; Lonza, Walkersville, MD) in a humidified atmosphere of 5% CO_2_. The immortalized HSAEC shows characteristics of normal cells such as contact inhibition of growth and failure to form soft agar colonies or form tumors in immune compromised mice. Immortalized human bronchial epithelial cells (HBEC) were grown in Keratinocyte serum-free medium (K-SFM, Life Technologies. Grand Island, NY) as described previously [33]. A549 cells were grown in RPMI supplemented with 5% fetal bovine serum. For induction of EMT, cells were TGF*β* treated for 5 d (10 ng/mL, R&D systems, Minneapolis, MN) in the growth medium.

### 2.5. Dual Cross-Link Chromatin Immunoprecipitation (XChIP)

XChIP was performed as described previously [34, 35]. A549 cells (~6 × 10^6^ per 100-mm dish) were washed twice with PBS. Protein-protein cross-linking was first performed with disuccinimidyl glutarate (2 mM, Pierce), followed by protein-DNA cross-linking with formaldehyde. Equal amounts of sheared chromatin were immunoprecipitated overnight at 4°C with 4 *μ*g of the indicated Ab in ChIP dilution buffer. Immunoprecipitates were collected with 40 *μ*L protein A magnetic beads (Dynal Inc.), washed, and eluted in 250 *μ*L elution buffer for 15 min at room temperature. Samples were de-cross-linked in 0.2 M NaCl at 65°C for 2 h. The precipitated DNA was phenol-chloroform extracted, precipitated with 100% ethanol, and dried.

Gene enrichment in XChIP was determined by quantitative real-time genomic PCR (Q-gPCR) as previously described [34] using region-specific PCR primers (Table 1). Standard curves were generated using a dilution series of genomic DNA (from 1 ng to 100 ng) for each primer pair. The fold change of DNA in each immunoprecipitate was determined by normalizing the absolute amount to the input DNA reference and calculating the fold change relative to that amount in unstimulated cells.

### 2.6. Deterministic Mathematical Modeling and Simulations

The deterministic mathematical model for both canonical and non-canonical arms of NF-*κ*B pathway is recently published where we have shown that TRAF1·NIK acts as a central, rate-limiting feed-forward signaling complex to activate noncanonical pathway [24]. The complete model consists of 28 ordinary differential equations (ODEs) and 58 parameters. The expression of genes in the canonical pathway is RelA-dependent, whereas, the noncanonical pathway genes are p52-dependent. We observed that there is a time delay in TRAF1 translation by nearly 120 min. Likewise, a delay of 90 min between NF*κ*B2 mRNA expression and translation has been reported earlier [36]. To examine the functional significance of these time delays in noncanonical pathway coupling, we performed different sets of simulations. In each simulation, the time delay function of TRAF1 and NF*κ*B2 translation was altered by the addition or subtraction of 15 min on either side of nominal value, while keeping the translation rate the same in each simulation. These simulations covered the time delay span of 0–10 h for both TRAF1 and NF*κ*B2 to study the effects of increasing and decreasing translation times on noncanonical pathway gene expression. In one set of simulations, we perturbed the system by varying the time-delay function of either TRAF1 or NF*κ*B2 one at a time and keeping all other rate constants at nominal values. In another set, we perturbed the system by varying these time-delay functions of both TRAF1 and NF-*κ*B2 simultaneously while keeping all other rate constants at nominal values. While the first set generated 42 such simulations for each protein, the second set generated more than 1600 such simulations. The latter will determine the synergistic effect of TRAF1 and NF-*κ*B2 on the gene expression of the noncanonical pathway.

### 2.7. Clustering Approaches

A clustered image map (CIM) of a normalized matrix was created that correlates gene expression pattern to different time points in HSAECs cells upon stimulation by TNF*α* and TGF*β*. For each gene, mean and standard deviation were calculated from their expression fold changes (see Table S1 in Supplementary Material available online at http://dx.doi.org/10.1155/2013/505864) across the time course and were normalized to unstimulated HSAECs [26–28]. *Z*-score transformation was calculated for each of the 50 genes (Table S2) by subtracting each fold change value by the row mean and dividing by the row standard deviation [37]. In essence, the *Z*-score gives an estimation of the deviation of the measurement from the row mean in standard deviation units. Hierarchical clustering was performed using an average-linkage clustering algorithm across six different time points in the absence or presence of stimulants. The cluster tree of genes is represented on the *y*-axis, and time-points and stimulants are shown on the *x*-axis. Each block of red or green represents a high positive or negative correlation between the gene expression and the stimulant under a specific time point.

## 3. Results and Discussion

### 3.1. Induction of Type II EMT

To establish a model of type II EMT, a continuously replicating line of human small airway epithelial cells was generated by immortalization using human telomerase (hTERT) and CDK4 expression [33]. These cells show a stable epithelial morphology and differentiated cytokeratin isoforms after over 100 population doublings, express the stem cell marker p63 and high levels of p16INK4a, and have an intact p53 checkpoint pathway [33].

**Figure 1:**
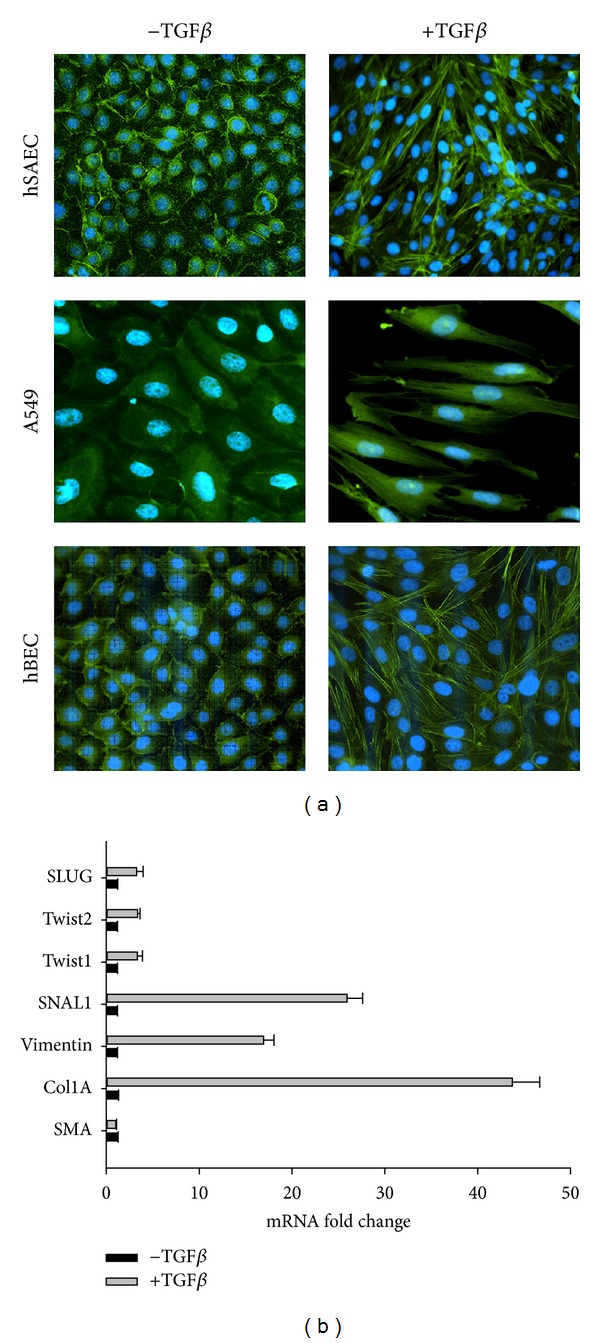
TGF*β* induces EMT transition in HSAECs. (a) F-actin staining. Shown in green is a confocal microscopic image of FITC-phalloidin staining merged with DAPI staining (blue) of HSAECs, A549 cells, and HBECs in the absence or presence of TGF*β* stimulation as indicated. (b) Expression of EMT program. Shown is normalized mRNA expression in HSAECs in the presence or absence of EMT after 10 d of TGF*β* treatment. For each gene, mRNA expression was normalized to *β*-pol as a housekeeping gene and expressed as the fold change relative to its expression in the absence of TGF*β* stimulation. Shown is mean and SE of replicate measurements. Each point is the mean of a duplicate biological experiment, measured with three technical replicates. Abbreviations: SLUG, Snail 2; SNAL1, Snail 1; col1A, collagen type 1a.

To characterize type II EMT, HSAECs were incubated in the absence or presence of TGF*β* (5 ng/mL) for 10 d. Transformed type II alveolar epithelial cells (A549) and immortalized bronchial epithelial cells (HBECs) were used as reference. Cells were fixed, stained with FITC-conjugated phalloidin (for distribution of F actin) and DAPI (a nuclear DNA stain), and examined by confocal microscopy. In the absence of TGF*β* stimulation, HSAECs assumed a normal cuboidal morphology with perinuclear cytoplasmic distribution of F-actin (Figure 1(a)). By contrast, TGF*β*-treated HSAECs showed an elongated shape with markedly induced F-actin staining (Figure 1(a)). This morphological change of enhanced front-rear polarity and cytoskeletal actin rearrangement are similar to those observed in TGF*β*-treated A549 and HBECs; all are characteristic morphological changes of EMT [8].

To further confirm the induction of EMT, expression of ECM genes and EMT-associated transcription factors were assessed by Q-RT-PCR. TGF*β*-treated HSAECs showed marked upregulation of extracellular matrix (ECM; Col1A), mesenchymal intermediate filament protein (vimentin), and TGF*β*-induced transcription factors (SNAI1, Twist1/2) mediating the EMT genetic program (Figure 1(b)). This gene expression pattern is similar to those observed in other primary epithelial cells [13]. Together, these data suggest that TGF*β* induces morphological and gene signatures of stable type II EMT in HSAECs.

### 3.2. Systems Profiling

**Figure 2:**
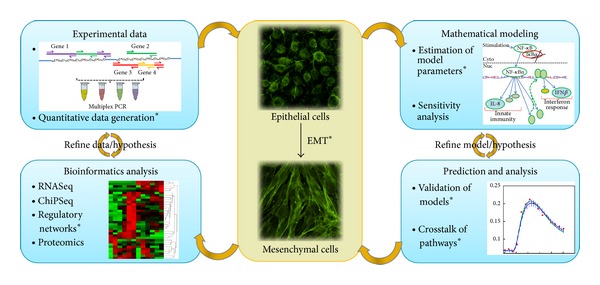
Systems approach to understanding EMT phenotypes. Schematic diagram of approach to understanding EMT phenotype using profiling and bioinformatics inferences. *indicates all the techniques/concepts that have been used in the current study, whereas others will be conducted in future studies.

Our goal was to establish a platform for the systematic perturbation of the signaling phenotype induced by stable type II EMT using reiterated rounds of stimulus perturbations and profiling measurements to inform the development of predictive models of complex behavior (schematically illustrated in Figure 2). For these experiments we developed a quantitative plate-based PCR assay to monitor the signature gene expression of the innate pathway, including the interferon, canonical and noncanonical NF-*κ*B dependent pathways, the DNA damage response pathways, the EMT programs, JAK/STAT pathway, and growth factor pathways (Table 1).

### 3.3. Perturbations of Signaling Dynamics in Response to EMT

**Figure 3:**
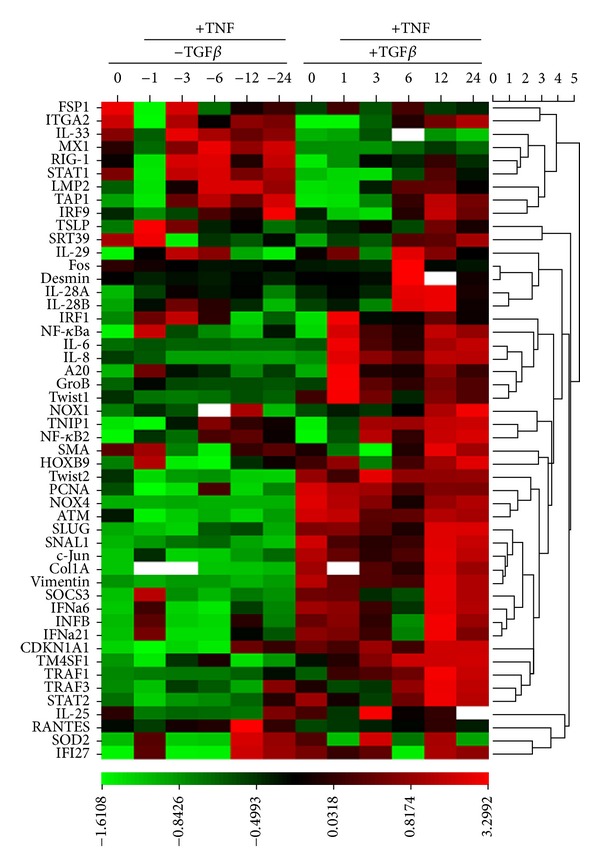
Effect of EMT on signaling pathways. (a) Heat map of gene expression. A time series of the TNF*α* stimulation of the HSAECs stimulated in the absence or presence of TGF*β*-induced type II EMT. Data are *Z*-score transformed and relative to unstimulated normal HSAECs and expressed as standard deviation (SD) units from the row mean. Scale for SD deviation is shown at the bottom. Gene abbreviations are shown in Table 1. The white rectangles represent the missing data where the fold changes and *Z*-scores could not be calculated.

The time series experiments of the gene expression driven by the canonical NF-*κ*B pathway were measured by using plate-based Q-RT-PCR in control and EMT-transformed HSAECs in response to the prototypical activating cytokine, TNF*α*. Relative changes in mRNA expression were normalized to DNA polymerase *β* as a housekeeping gene, and the data were *Z*-score-transformed to show deviations of expression in standard deviation units [26, 37]. The gene expression of the pathway was then visualized using hierarchical clustering (Figure 3, and Supplemental Tables). The HSAECs after EMT showed unique quantitative and qualitative expression patterns. For example, the top cluster contains members of the interferon-gamma (IFN) pathway such as ITGA2, myxovirus resistance 1 (MX1), signal transducer and activator of transcription (STAT1), proteasomal components low molecular weight proteins (LMP2), and transporters associated with antigen processing (TAP2); these genes are markedly downregulated as a function of EMT (Figure 3). By contrast, genes within the NF-*κ*B pathway, growth factor response, and EMT signatures were markedly upregulated (Figure 3).

### 3.4. EMT Effects on the Canonical NF-*κ*B Signaling Pathway

**Figure 4:**
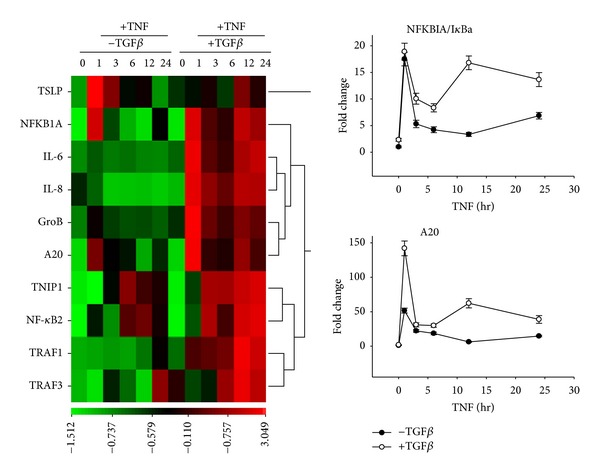
Dysregulation of canonical NF-*κ*B signaling in EMT. Left panel, heat map of NF-*κ*B selective reporter genes. Data are *Z*-score-transformed and relative to unstimulated normal HSAECs and expressed as standard deviation (SD) units from the row mean. Scale for SD deviation is shown at the bottom. Right panel, quantitation of NFKBIA/I*κ*Ba and TNFAIP3/A20 gene expressions in HSAECs and EMT-HSAECs as indicated. Shown is fold change mRNA relative to unstimulated normal HSAECs. Each point is the mean of a duplicate biological experiment, measured with three technical replicates.

Because of the marked effects of the EMT state on the NF-*κ*B pathway, we sought to further investigate this mechanism. Hierarchical clustering of the NF-*κ*B-dependent gene pathway is shown in Figure 4. Genes that selectively respond to the canonical pathway include NFKBIA/I*κ*Ba and TNFAIP3/A20; we have shown that the expression of these genes depends on I*κ*B*α* proteolysis and that they have direct NF-*κ*B/RelA binding sites in their promoters [26, 38]. Greater upregulation of these genes was observed at the earliest time point measured, with a second wave of expression at 12–24 h after TNF*α* stimulation. This mimics the oscillatory behavior of the NF-*κ*B signaling pathway observed in cancer cells [39].

### 3.5. EMT Affects the Coupling of the Noncanonical to the Canonical NF-*κ*B Signaling Pathways

**Figure 5:**
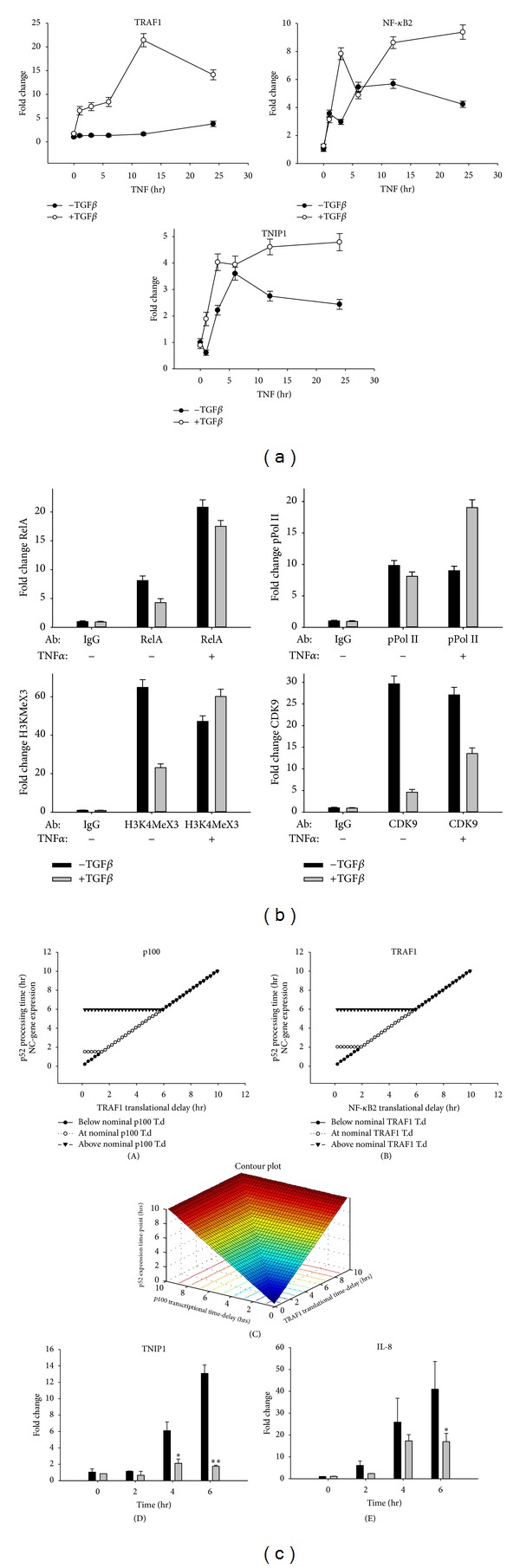
Effect of EMT on canonical-noncanonical NF-*κ*B pathway coupling. (a) Relative changes in TRAF1, NF-*κ*B2, and TNIP1 mRNA expressed as fold-change measurements in the absence or presence of TGF*β*-induced EMT as indicated. Each point is the mean of a duplicate biological experiment, measured with three technical replicates. (b) XChIP experiments of HSAECs in the presence (grey bars) or absence (black bars) of TGF*β*-induced EMT. Shown is fold change in the TRAF1 promoter quantified by Q-gPCR relative to unstimulated HSAEC signal in duplicate experiments. (c) Computational simulations of p52 processing as a function of translational delay for TRAF1 and NF*κ*B2. Abbreviations; T.d., translational delay. (A) shows the effect of increasing TRAF1 translational delay on p52 processing time while keeping the translational delay of NF*κ*B2 either at nominal rate (90′) or higher than nominal rate or lower than nominal rate. (B) shows similar effect but for increasing NF*κ*B2 translational delay (*x*-axis). (C) shows the contour plot of all simulations (D, E) amantadine-treated A549 cells (200 *μ*g/mL) were stimulated with poly I:C and TNIP1/Naf1 (D), and IL8 (E) expression was measured by Q-RT-PCR. Data expressed as fold change as compared to untreated cells after normalizing to internal controls, GAPDH. Data analyzed by a 2-way ANOVA with multiple comparisons. Significantly different from amantadine untreated samples: *P < 0.05 and **P < 0.001. Amantadine-treated cells (light bars) showed higher level of noncanonical pathway inhibition compared to untreated cells (dark bars).

EMT effects on the time-dependent expression of the TNIP1/Naf1 gene, a hallmark of the noncanonical NF-*κ*B pathway [24, 27], were also evident in the hierarchical clustering (Figures 3 and 4). As discussed above, the noncanonical pathway is coupled to the canonical pathway through the expression of TNF receptor associated factor-1 (TRAF-1). TRAF-1 is unique for the TRAF isoforms that complexes with, stabilizes, and activates the NIK·IKK*α* complex to trigger the noncanonical pathway [24]. Analysis of relative fold change of expression showed that TRAF-1 expression was strongly upregulated within 1 h of TNF*α* stimulation and peaked at 12 h in EMT-HSAECs versus HSAECs, whereas, TRAF-1 was upregulated by 20-fold at 12 h after TNF*α* stimulation in EMT-HSEACs versus 3-fold in HSEACs (Figure 5(a)). 

We especially noted that the noncanonical NF-*κ*B pathway-dependent TNIP1/Naf1 gene showed a leftward shift in the temporal expression profile, where, in the presence of EMT, TNIP1/Naf1 expression was more rapidly induced, reaching the plateau within 3 h of stimulation in the EMT-HSAECs versus the 6 h required to reach plateau seen in HSAECs (Figure 5(a)). Together, these data indicate that EMT induces more robust expression of TRAF1 and produced a rapid coupling of the canonical to the noncanonical NF-*κ*B pathway. 

### 3.6. EMT Effects on Chromatin Modification and Transcriptional Elongation

Transcription of protein-coding genes by RNA Pol II is a highly regulated process involving preinitiation (assembly of basal transcription factors and co-activator recruitment), leading to initiation, elongation, and termination phases of RNA synthesis [40]. Previous work by our group and others has shown that transcriptional elongation is a major regulated event controlling the innate response [38, 41, 42]. Because genome-wide analysis of EMT has revealed that TGF*β* induces global reorganization of transcriptionally active marks (histone (H3) Lys (K) 36 trimethyl) in large organized heterochromatin domains [20], we examined whether the NF-*κ*B-dependent genes were affected by chromatin remodeling or via changes in transcriptional elongation.

We therefore measured the binding of the transactivator (NF*κ*B/RelA), transcriptional elongation complex (CDK9), activated transcriptional elongation polymerase (phospho-Ser^2^ RNA Pol II), and euchromatin marks (H3K4Me3) on the TRAF-1 promoter using a highly quantitative two-step chromatin immunoprecipitation (XChIP) assay [34, 43]. Here, TNF*α* induced a similar level of increase in NF-*κ*B/RelA binding to promoter in both EMT-HSAECs and normal HSAECs. This induction of NF-*κ*B/RelA binding was not affected by EMT (Figure 5(b)). TNF*α* stimulation induced a significant increase in phospho-Ser^2^ RNA Pol II binding in the EMT-HSAECs, despite reduced amounts of CDK9 binding in both basal and TNF*α* stimulated conditions (Figure 5(b)). EMT induced the level of H3K4Me3 binding to the TRAF1 promoter, even in the absence of TNF*α* stimulation. Together, these findings indicate that EMT promotes remodeling of the chromatin environment of the TRAF1 gene, poising it for more rapid induction via transcriptional elongation. 

### 3.7. Computational Simulations of the Effect of EMT on Canonical-Noncanonical NF-*κ*B Coupling

We previously reported a deterministic mathematical model linking the canonical and noncanonical arms of NF-*κ*B pathway, where we have shown that the formation of the TRAF1·NIK complex acts as a rate-limiting feed-forward signaling complex necessary to activate the noncanonical pathway [24]. 

Based on the time delay between the expression of TRAF1 mRNA and TRAF1 protein and the presence of an atypical internal ribosomal binding site (IRES) and translational initiation site at an internal site in TRAF1 [44], we predicted that a delay in TRAF1 protein translation via a cap-independent mechanism controls the rate of noncanonical NF-*κ*B pathway activation in response to TNF*α*. Interestingly, our expression profiling data here suggest that pathway coupling is affected by EMT in the HSAECs. Through the enhanced formation of transcriptional elongation-competent RNA Pol II, TRAF1 and NF-*κ*B2 expression is enhanced, resulting in a shift of noncanonical pathway activation (TNIP1/Naf1) to earlier times. 

We, therefore, performed simulations of the noncanonical pathway coupling by systematically varying the translation delay function of TRAF1. Interestingly, the coupling interval, that is, time lag between the activation of canonical NF-*κ*B pathway and the formation of p52, monotonically reduced as TRAF-1 translation was increased until it reached a critical threshold of 90 min (Figure 5(c): (A)). Further reductions in the TRAF-1 translation delay function alone, even to zero, did not reduce the coupling interval for p52 formation and TNIP/Naf1 expression. 

After examination of other proteins in the noncanonical pathway, we noted that NF-*κ*B2 translation delay function produced a similar effect, where the coupling interval reached a plateau below which p52 processing was not further increased (Figure 5(c): (B)). In these experiments, a total of 42 simulations were conducted for each protein covering the time points from zero to 10 h.

The finding that the coupling interval was inert to reducing the translational delay of each protein individually prompted us to perform a simulation by changing the translational delay parameters for both TRAF1 and NF-*κ*B2 simultaneously. The biological rationale for this is that TRAF-1 is translated by a cap-independent mechanism [44] and our previous studies show that both of these mRNAs are translated in a coordinated, delayed manner distinct from those mRNAs undergoing cap-dependent translation. A total of 1681 combinatorial simulations of the translational delay were conducted covering the time points from 0 to 10 h. Interestingly, we found that reducing the IRES-dependent translational delays for both proteins together resulted in shorter coupling interval (e.g., an earlier onset of p52 processing and noncanonical gene expression) to values as quickly as 15 min relative to TNF*α* stimulation (versus the nominal time of 6 h) (Figure 5(c): (A), (B)). In both figures, reducing the translational delay of both proteins to zero resulted in the early processing of p100 to p52. Figure 5(c): (C) shows the cumulative effect of all combinations of simulations for these two proteins. A conceptual representation is shown in Table 2. This model thus predicts that altering the translation rate of these two proteins produces a leftward shift in noncanonical gene expression kinetics relative to the canonical pathway. 

**Figure 6:**
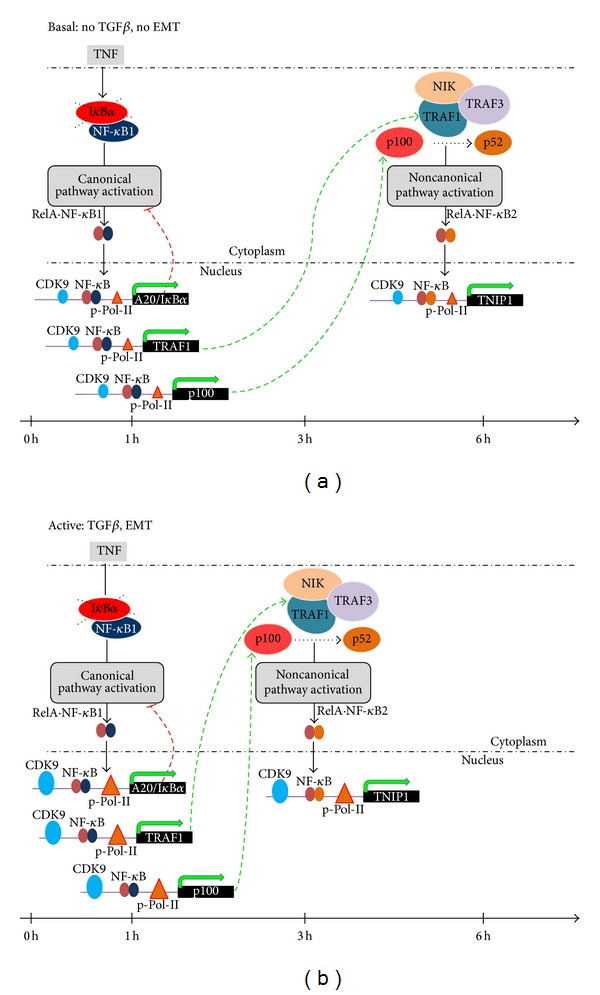
Effect of EMT on canonical-noncanonical NF-*κ*B coupling. Schematic diagram of EMT effects on NF-*κ*B signaling pathway. EMT induces changes in euchromatin marks of NF-*κ*B-dependent genes, transcriptional elongation, and translational delay to reduce the coupling constant between the NF-*κ*B canonical and noncanonical pathways. A timeline is shown for both states of EMT-Basal (no EMT) and TGF*β*-mediated EMT (0 to 6 h).

Our computational model predicted that TRAF1 and p100 translation is under IRES-dependent control. Our earlier studies have shown that the activation kinetics of noncanonical pathway is coincident with that of the canonical pathway in response to viral infection and dsRNA stimulation [45]. We noted that the effects of dsRNA are similar to that of EMT by reducing the coupling constant between the canonical and noncanonical pathways. To confirm whether the noncanonical pathway was coupled to NF-*κ*B/RelA through an IRES dependent mechanism, we perturbed an IRES mediated translation using amantadine, a known inhibitor of IRES-dependent translation [46]. A549 cells were activated using poly I:C treatment in the absence or presence of amantadine, and canonical and noncanonical pathway kinetics were measured. We observed that the amantadine-treated cells showed significant inhibition of TNIP1/Naf1 expression compared to control cells (no amantadine treatment) after 4 and 6 h of treatment (Figure 5(c): (D)). At this dose, amantadine seems to have some inhibitory effect on IL8 expression at 6 hr (Figure 5(c): (E)) although this effect was significantly lower than its effect on TNIP1/Naf1 (7-fold versus 2-fold) (Figure 5(c): (D), (E)). These results suggest that EMT results in chromatin remodeling and transcriptional elongation of TRAF-1/NF*κ*B2 expression as well as influencing the rate of translation of IRES-dependent genes, resulting in shortening the coupling interval between the canonical and noncanonical pathways (Figure 6). 

## 4. Discussion

In this study, we applied systems level approaches to interrogate the dynamic state of EMT in a model of chronic mucosal inflammation. Although EMT processes (type I EMT) are essential during histogenesis and organogenesis, type II EMT is involved in organ remodeling and chronic diseases, and type III EMT is involved in progression of transformed epithelial cells to a metastatic phenotype. These newly formed mesenchymal cells transiently express distinct markers acquiring “front-rear” polarity, become invasive, and induce extracellular matrix remodeling. Using immortalized human small airway epithelial cells, we demonstrate that TGF*β* induces stable EMT transition morphologically through the restructuring of actin cytoskeleton, induction of intermediate mesenchymal cytoskeletal proteins, and expression of the SNALI/Twist transcription factors. Although the signals conferring EMT have been extensively investigated, the effect of EMT on signal transduction pathways has not been systematically explored. Here, we investigated the effect of EMT on innate signaling and discovered that EMT dramatically affects two coupled arms of the NF-*κ*B signaling pathway. This coupling is mediated through the induction of transcriptionally active euchromatin marks and enhancement of transcriptional elongation. Our computational simulations further predict that translational rate of these two key rate-limiting coupling proteins must also be affected. As a result, the innate pathway is hyperresponsive in type II EMT, with a more rapid coupling between the canonical and noncanonical arms of the NF-*κ*B pathway. Because EMT has been observed in chronic lung fibrotic disease in humans [47] and epithelial cells isolated from human asthmatics undergo a greater degree of EMT than normal subjects [13], these studies have special relevance to airway pathophysiology.

Type II EMT may play a critical cellular role in the progression of human pulmonary fibrotic diseases. Although the molecular basis of airway remodeling and loss of epithelial integrity in asthma is still undefined, several lines of evidence implicate EMT in this process [48]. For example, the airway epithelium is recognized as an important contributor to intrapulmonary fibroblast accumulation after injury, including idiopathic pulmonary fibrosis [12, 49], asthma, and COPD [50]. The bronchial epithelium in asthma is highly abnormal with structural changes involving the separation of columnar cells from their basal attachment leading to the disruption of epithelial barrier and functional changes including increased expression and release of profibrotic factors [2]. Subepithelial fibrosis is influenced by epithelial cell-derived VEGF, IL-33, IL-25, and thymic stromal lymphopoietin (TSLP), as well as leukocyte-derived TGF*β*. Epithelial cells from individuals with asthma produce high amounts of periostin, a cytokine that stimulates TGF-*β* production and modifies myofibroblast collagen synthesis [51]. The increase in myofibroblasts causes thickening and increased density of the subepithelial basement membrane, contributing to a progressive decline in lung function [48, 52]. 

The relationship between epithelial fibrosis and innate inflammation is a complex and interrelated phenomenon. Specifically, the TNF*α*-NF-*κ*B pathway has a number of modulatory actions on epithelial EMT. For example, activated NF-*κ*B suppresses E-Cad and induces the expression of mesenchymal vimentin, two hallmarks of EMT [53]. Studies of insulin breast cancer epithelial cells have shown that like growth factor (IGF)1 receptor-induced EMT is partially mediated by NF-*κ*B-increased expression of SNAL1 [54]. SNAI1 upregulation is mediated at multiple levels, including direct binding to the SNAI1 promoter leading to an increased mRNA expression at the level of transcription [55] and secondly by induction of CSN2, a protein that disrupts binding GSK-3*β* and *β*-Trcp binding to SNAI1 resulting in its posttranslational stabilization [56]. Our observations suggest that EMT-HSAECs express high levels of SNAI1, and that upregulation of NF-*κ*B is partially responsible for mediating type II EMT. More work will be required to determine the requirement of NF-*κ*B signaling in EMT of HSAECs. We note that other studies have shown that NF-*κ*B activates ZEB in mammary epithelial cells through cis-regulatory elements in the ZEB promoter [53], perhaps suggesting that NF-*κ*B is a master regulator of EMT programs. 

An exciting and novel finding in our studies is that EMT sensitized the innate pathway to be hyperresponsive to its canonical activating signals. Specifically, we demonstrate that EMT dramatically upregulates innate genes and accelerates the coordinated induction of TRAF1 and NF-*κ*B2 expression by modulating euchromatin marks and inducing transcriptional elongation. Our XChIP experiments indicate that genes downstream of the NF-*κ*B pathway are regulated by a mechanism involving enhanced formation of phospho-Ser^2^ Pol II. The NF-*κ*B pathway is a major arm of the innate immune response, whose rapid activation generates effectors that restrict pathogen spread [23, 57]. For innate genes bounded within closed chromatin, preinitiation complex formation is an essential first step in inducible expression [40]. In this process, the histone acetyltransferases, such as p300/CBP, are recruited to destabilize repressive histone, resulting in p300/CBP dissociation the and binding of general transcription factors, including TFIID. By contrast, for immediate early genes located within open chromatin, Pol II is preengaged in a hypophosphorylated state, producing short ~30–50 nt transcripts [58]. Promoter proximal pausing is reversed by the activated positive transcription elongation factor (P-TEFb), a multiprotein complex containing CDK9, BRD4 and cyclin T1 or T2 subunits [41, 42]. Upon PTEF-b binding, phospho-Ser^2^ Pol II is formed, and enters elongation mode to produce full-length, spliced transcripts [41, 42, 59]. Our XChIP analysis demonstrates that innate genes in EMT-HSAECs are located within an euchromatin environment, associated with transcriptionally active histone H3 marks. This finding is consistent with RelA binding preferring open chromatin domains, inferred by our earlier ChIP-Seq studies [43]. The effect of EMT on enhanced formation of phospho-Ser^2^ RNA Pol II has not been previously observed and will require further investigation into the effects of EMT on the PTEF-b protein interaction network. 

Our data further indicate that EMT affects the feed-forward link between the canonical and noncanonical NF-*κ*B pathways. Earlier, we showed that TRAF1 and NF-*κ*B2 are canonical genes whose protein synthesis is under a translational delay [24]. Newly synthesized TRAF1 binds and stabilizes NIK by disrupting its interaction with TRAF2-cIAP2. Activated NIK is the rate limiting step responsible for the co-translational processing of NF-*κ*B2 precursor into the active 50 kDa DNA binding form [24]. Our simulations predict that EMT controls the rate of IRES-dependent translation of both TRAF1 and NF-*κ*B2 to explain this effect. The amantadine inhibition experiments validate these predictions; however, more research will be required to understand the effect of EMT on IRES-dependent translational control. 

We contend that the systematic examination of type II EMT of airway epithelial cells has important implications for human disease. Recent studies suggest that the structural components of the lung may first respond to environmental risk factors for inflammatory/fibrotic lung diseases, such as asthma [60], COPD, and cancer. In asthma, EMT remodeling of the epithelial basement membrane promotes sensitization to inhaled allergen by causing persistent dendritic cell activation and migration [61]. In COPD, the reticular basement membrane is fragmented with “clefts” of cells staining for MMP-9 and S100A4, hallmarks of EMT [13]. We note that airways infection with respiratory syncytial virus (RSV), the most common viral respiratory pathogen in small children which is widely considered as a risk factor for the development of allergic asthma later in life is characterized by an increased expression of SNAI1, MMP-2, and TGF*β*1 [62, 63]. Finally, EMT plays a critical role in switching a primary tumor to a malignant cancer with metastatic phenotype. These data suggest that induction of EMT is seen in a number of lung pathologies.

In summary, using RNA profiling of an EMT cell model, we demonstrate that this transition has dramatic effects on the induction of the innate pathway through a mechanism involving chromatin remodeling of the TRAF1 locus, enabling enhanced phospho-Pol II loading and transcriptional elongation. Computational simulation experiments suggest that rapid, coordinate, TRAF1/NF*κ*B2 expression is coupled to their IRES-dependent translation. These rapidly expressed proteins produce a feed-forward mechanism that reduces the coupling interval between the canonical and noncanonical signaling arms of the NF-*κ*B pathways. Further applications of systems approaches will promote a comprehensive understanding of this complex phenotype through reiterated rounds of gene perturbation, multidimensional profiling, and deterministic modeling.

## Supplementary Material

Supplementary Information: Table S1 represents the fold change of all genes for a given time-course in EMT (+TGF) and non-EMT (-TGF) states of HSAECs. For genes whose expression value could not be measured, their fold change value is marked as NA. Table S2 represents the Z scores of all genes for a given time-course in EMT (+TGF) and non-EMT (-TGF) states of HSAECs. The method of Z score calculation is explained in the Methods section of the manuscript. This is a standard transformation that expresses the sample deviation from the row mean in standard deviation units, and allows gene expression data derived from different microarray studies to be comparable across experiments. For genes whose expression value could not be measured, their fold change value is marked as NA.

## Figures and Tables

**Table 1:** Primer sequences in Q-RT-PCR assay.

Pathway	Gene	Accession #	Amplicon (bp)	Forward (5′-3′)	Reverse (5′-3′)
Canonical	NFKB1A	NM_020529	116	CCGCAGGAGGTGCCG	ATCACTTCCATGGTCAGTGCC
	CXCL2/GroB	NM_002089	100	ATTCACCTCAAGAACATCCAAAGTG	GCCCATTCTTGAGTGTGGCTAT
	TNFA1P3/A20	NM_001270508	111	GAAGCACCATGTTTGAAGGATACTG	CTCTGCGCTGGCTCGATC
	IL-6	NM_000600	90	CTGGATTCAATGAGGAGACTTGC	TCAAATCTGTTCTGGAGGTACTCTAGG
	IL-8	NM_000584	92	AAGACATACTCCAAACCTTTCCACC	CAATAATTTCTGTGTTGGCGCA
	TSLP	NM_033035	91	TCTTGTAGCAATCGGCCACA	ACATTTCTTTGGCGAGCGA
	TRAF1	NM_005658	91	TGGAAGATCACCAATGTCACCA	ATACTTGGCAGTGTAGAAGGCTGG
	TRAF3	NM_145752	91	GAAGGCGTGTAAATACCGGG	ACAGTCGGTGTCTTCGTGTTTCT
	NFKB2	NM_001077494	96	ACATGACTGCCCAATTTAACAACC	GGAGCCGCTGCCTCTGA
	IL-25	NM_022789	91	CACCCAGAGTCCTGTAGGGC	GGTTCAAGTCTCTGTCCAACTCATATC
	IL-33	NM_033439	51	AACACCCCTCAAATGAATCAGGT	TTGGCATGCAACCAGAAGTCT

Noncanonical EMT	TNIP1	NM_001252385	92	ATCCAGTGGCACCTCCTCTG	CCAGCGCCATCGCATT
	SMA/ACTA2	NM_001141945	91	TGTAAGGCCGGCTTTGCT	TTCCCACCATCACCCCCT
	Col1A1	NM_000088	95	CCAGAAGAACTGGTACATCAGCA	CGCCATACTCGAACTGGAATC
	Vimentin	NM_003380	91	GCTCAATGTTAAGATGGCCCTT	TGGAAGAGGCAGAGAAATCCTG
	Desmin	NM_001927	91	GGAGAGGAGAGCCGGATCA	GGACCTCAGAACCCCTTTGC
	Twist1	NM_000474	101	TCTCGGTCTGGAGGATGGA	CAATGACATCTAGGTCTCCG
	Twist2	NM_001271893	119	ACGAGCGCCTCAGCTACG	CGCGACGGACAGCCCTG
	SLUG	AF084243	91	TGTGTGGACTACCGCTGCTC	ACTCACTCGCCCCAAAGATG
	SNAI1	NM_005985	95	GCGCTCTTTCCTCGTCAGG	GGGCTGCTGGAAGGTAAACTCT
	ITGA2	NM_002203	91	AGCCGAAGTACCAACAGGAGTTATA	GCCGAGCTTCCATAAAATTGC
	FSP1/S100A4	NM_002961	91	AGGGTGACAAGTTCAAGCTCAAC	GCTTCATCTGTCCTTTTCCCC
	SMA/ACTA2	NM_001141945	91	TGTAAGGCCGGCTTTGCT	TTCCCACCATCACCCCCT

IFN	IFNb	NM_002176	95	GCAGTTCCAGAAGGAGGACG	TCCAGCCAGTGCTAGATGAATC
	IFNa6	NM_021002	91	GTGGTGCTCAGCTGCAAGTC	CCAGGAGCATCATGGTCCTC
	IFNa21	NM_002175	91	TGATCTGCCTCAGACCCACA	CTTCAGGCAGGAGAAAGGAGAG
	CCL5/RANTES	NM_002985	96	TCTACACCAGTGGCAAGTGCTC	CCCGAACCCATTTCTTCTCTG
	DDX58/RIG1	NM_014314	132	CCACTTAAACCCAGAGACAATAACAA	TTGCCACGTCCAGTCAATATG
	LMP2	NM_002800	91	TTCACCACAGACGCTATTGCTC	CCACACCGGCAGCTGTAAT
	TAP1	NM_000593	91	GTTTTTCCAACAGAACCAGACAGG	GCTCAGATTCTCACTCAGAGAATCACT
	STAT1	NM_007315	104	TCCTGCTGCGGTTCAGTG	GGGTTCAACCGCATGGAAG
	STAT2	NM_005419	118	CCTGAAACACAGGCTCATTGTG	TGGCACCAGCCCTAGTTCC
	IRF1	NM_002198	91	AGCAAGGCCAAGAGGAAGTCA	TGCTGTGGTCATCAGGCAGA
	IRF9	NM_006084	91	AGCCACAGGAAGTTACAGACACAA	GCAGTGAGTAGTCTGGCTCTGGA
	SOCS3	NM_003955	91	CTTTCTGATCCGCGACAGCT	ACACTGGATGCGCAGGTTC
	IF127	NM_001130080	91	CAGTCACTGGGAGCAACTGGA	GCCCAGGATGAACTTGGTCA
	MX1	NM_001144925	106	GAACCACCCATATTTCAGGGATC	ATGTGTGATGAGCTCGCTGGTA
	IFNL1 (IL-29)	NM_172140	103	AAGCCCACCACAACTGGG	ACTCTTCCAAGGCGTCCC
	IFNL2 (IL-28A)	NM_172138	101	ACATGACTGGGGACTGCAC	ATCCGGGAGAGCCCCGT
	IFNL3 (IL-28B)	NM_172139	99	ATGACCGGGGACTGCATG	ATCCGGGAGAGCCCCGC

Growth factor	Fos	NM_005252	91	GGGCAAGGTGGAACAGTTATCT	GTTGCGGCATTTGGCTG
	c-Jun	NM_002228	91	GTCCCAGGAGCGGATCAAG	GGCGATTCTCTCCAGCTTCC
	SOD2	NM_000636	90	TGCTTGTCCAAATCAGGATCC	TGAAGGTAGTAAGCGTGCTCCC
	TM4SF1	NM_014220	91	GAGGTGGCCTGCTGATGCT	CCACAGTTTTCATGGCCACAG

ROS/DNA	NOX1	NM_007052	91	GGGCATCCCCCTGAGTCT	TCTGCTGGGAGCGGTAAAAC
	NOX4	NM_016931	91	ACTCAACACCCTGTTGGATGACT	CCAACGGAAGGACTGGATATCT
	HOXB9	NM_024017	91	GGCCGGATCAAACCAACC	TCCAGCGTCTGGTATTTGGTG
	ATM	NM_000051	127	GCTTCTCAGGATAATCCGCAAG	CCAAGCAGCTTCCAACAGC
	STK39 (SRT39)	NM_013233	91	GCCCAAAGAGCCAAAAAGGT	CGTCGTCACTCCACTCCCA

Cell cycle	PCNA	NM_002592	91	TGCGCCGGCAATGAA	CTTTCTCCTGGTTTGGTGCTTC
	CDKN1A	NM_000389	92	AGCAGGCTGAAGGGTCCC	GCGTTTGGAGTGGTAGAAATCTGT

**Table 2:** Conceptual representation of combinatorial simulations of translational delays of TRAF1 and NF-*κ*B2.

Time-delay combinations (min)	NF-*κ*B2/p100 processing kinetics
(1) TRAF1 (<120) + P100 (<90)	Fast

(2) TRAF1 (≤120) + P100 (=90) OR TRAF1 (=120) + P100 (≤90)	Near nominal or nominal

(3) TRAF1 (≥120) + P100 (>90) OR TRAF1 (>120) + P100 (≤90) OR TRAF1 (≤120) + P100 (>90)	Delayed
