# Supplementary material for: Systems Approaches to Modeling Chronic Mucosal Inflammation
Source: Biomed Res Int. 2013 Oct 21;2013:505864. doi: 10.1155/2013/505864 (PMC3818818; doi:10.1155/2013/505864)
Supplement: Supplementary file 1 — Supplementary Information: Table S1 represents the fold change of all genes for a given time-course in EMT (+TGF) and non-EMT (-TGF) states of HSAECs. For genes whose expression value could not be measured, their fold change value is marked as NA. Table S2 represents the Z scores of all genes for a given time-course in EMT (+TGF) and non-EMT (-TGF) states of HSAECs. The method of Z score calculation is explained in the Methods section of the manuscript. This is a standard transformation that expresses the sample deviation from the row mean in standard deviation units, and allows gene expression data derived from different microarray studies to be comparable across experiments. For genes whose expression value could not be measured, their fold change value is marked as NA. [file 505864.f1.pdf]

Table S1: Fold change of all genes for a given time-course in EMT and non-EMT states of cells.

	- TGFβ						+ TGFβ					
TNFα→	Ctl	1hr	3Hr	6Hr	12Hr	24Hr	Ctl	1hr	3Hr	6Hr	12Hr	24Hr
NFKBa	1.0	17.509	5.315	4.228	3.340	6.869	2.430	18.896	10.056	8.340	16.795	13.642
GroB	1.0	12.817	3.972	2.789	2.888	2.028	5.054	116.162	32.447	22.471	38.586	30.910
TNIP1	1.0	0.610	2.220	3.600	2.750	2.440	0.890	1.890	4.030	3.940	4.610	4.790
IL-6	1.0	70.035	26.538	32.900	1.828	1.636	49.199	3420.520	1052.789	675.588	1833.011	2194.992
IL-8	1.0	0.620	0.054	0.073	0.093	0.053	0.228	8.340	4.627	3.411	6.233	6.021
TSLP	1.0	13.454	5.134	2.144	2.567	1.057	1.529	2.114	2.713	1.464	4.993	3.010
TRAF1	1.0	1.275	1.347	1.329	1.636	3.784	1.707	6.543	7.413	8.398	21.407	14.123
A20	1.0	51.625	22.162	18.507	6.148	14.621	1.818	142.025	30.910	29.857	62.250	38.854
TRAF3	1.0	0.812	1.320	1.149	0.908	2.732	1.800	1.223	1.347	2.828	4.724	3.434
INFB	1.0	4.377	0.758	0.540	3.411	1.505	4.676	5.464	3.580	1.376	10.411	5.816
IL-25	1.0	0.006	0.057	0.042	0.012	1.853	1.326	0.334	5.098	0.702	1.110	NA
IL-33	1.0	0.191	1.905	1.189	0.841	1.028	0.052	0.072	0.206	NA	0.096	0.003
IFNa6	1.0	3.506	0.835	0.480	2.028	1.474	5.046	4.757	3.434	2.085	7.835	5.502
IFNa21	1.0	4.993	0.543	0.547	2.809	1.815	5.587	5.169	4.000	1.537	10.411	5.242
RANTES	1.0	0.330	1.526	1.729	12.817	2.173	0.097	0.384	0.801	1.087	2.071	0.807
RIG-1	1.0	0.339	2.282	2.412	1.094	2.056	0.312	0.603	0.946	0.889	1.197	0.853
LMP2	1.0	0.230	1.729	4.532	4.408	3.117	0.244	0.392	1.424	2.428	2.479	1.591
TAP1	1.0	0.559	2.428	3.364	2.428	3.784	0.569	0.611	1.110	2.056	3.411	2.549
STAT1	1.0	0.423	1.240	1.395	0.979	1.117	0.519	0.470	0.457	0.624	0.908	0.707
STAT2	1.0	0.590	0.841	0.895	1.021	1.828	3.038	1.705	1.214	2.532	5.098	3.458
IRF1	1.0	2.908	4.287	2.219	0.566	1.173	0.373	6.148	1.741	1.879	2.828	1.892
IRF9	1.0	0.732	0.877	1.444	1.189	3.031	1.021	0.603	0.521	1.231	2.204	1.569
SOCS3	1.0	4.112	1.214	1.042	1.310	1.189	3.046	2.969	1.729	1.474	5.579	3.918
Fos	1.0	0.582	0.409	0.210	0.232	0.363	0.184	0.141	0.051	8.815	0.304	0.191
c-Jun	1.0	2.657	0.693	0.901	1.424	1.879	7.543	5.352	4.056	4.925	10.778	8.112
SOD2	1.0	12.553	0.310	0.503	25.813	17.388	12.401	0.966	24.590	2.990	19.027	1.682
NOX1	1.0	2.282	1.404	NA	7.945	0.255	1.537	2.479	1.959	2.908	8.056	13.737
NOX4	1.0	0.323	38.055	0.140	0.595	0.470	4977.644	3565.775	2797.650	1360.574	3040.304	3516.684
FSP1	1.0	0.196	0.829	0.339	0.451	0.518	0.375	0.525	0.354	0.529	0.382	0.406
SMA	1.0	1.214	0.629	0.435	0.920	0.993	0.852	0.637	0.332	0.859	1.659	1.214
Col1A	1.0	NA	NA	0.871	1.173	2.313	43.701	NA	29.857	22.009	68.120	42.814
Vimentin	1.0	0.438	0.889	0.889	0.387	0.883	16.800	10.629	9.781	9.000	25.281	16.223
Desmin	1.0	0.003	0.435	0.137	0.707	0.004	0.923	0.966	0.457	27.474	NA	1.474
MX1	1.0	0.193	1.840	3.784	1.986	2.908	0.076	0.056	0.047	0.206	0.423	0.199
IFI27	1.0	28.443	1.141	1.301	48.168	37.271	31.722	25.281	29.243	1.905	40.224	35.753
NFKB2	1.0	3.560	2.970	5.460	5.690	4.230	1.250	3.140	7.840	4.890	8.630	9.380
CDKN1A1	1.0	0.090	1.000	0.680	3.810	3.160	3.810	3.390	4.590	3.320	5.860	5.810
PCNA	1.0	0.480	0.710	1.460	0.760	0.920	2.280	1.910	1.820	1.440	1.670	1.640
ITGA2	1.0	0.250	0.900	0.570	0.810	0.770	0.290	0.290	0.460	0.610	0.750	0.890
SRT39	1.0	1.270	0.590	0.740	0.710	0.770	0.670	0.730	0.710	0.880	0.890	1.000
TM4SF1	1.0	0.540	1.380	1.800	0.610	1.000	1.560	1.850	2.680	3.610	3.610	3.640
ATM	1.0	0.390	0.590	0.660	0.540	0.690	2.210	2.060	1.420	1.450	1.930	1.850
IL-28A	1.0	2.850	4.380	3.780	4.110	1.820	3.560	4.140	3.510	15.900	16.200	4.890
IL-28B	1.0	2.390	4.350	3.110	2.160	0.860	1.760	2.310	1.240	7.940	9.250	2.690
IL-29	1.0	2.990	5.780	4.720	1.850	1.070	3.010	4.350	2.010	7.160	4.630	2.970
HOXB9	1.0	2.680	0.510	0.290	1.270	1.490	1.830	2.380	1.000	1.840	2.490	3.510
SLUG	1.0	0.920	0.770	1.260	1.350	1.000	2.830	2.910	2.430	1.950	4.350	4.320
SNAL1	1.0	1.790	2.930	3.210	1.450	0.730	25.400	12.600	9.600	11.200	29.200	23.800
TWIST1	1.0	0.330	0.290	0.340	0.570	0.440	2.730	10.300	3.660	1.210	4.030	3.050
TWIST2	1.0	0.120	0.490	0.520	0.250	0.190	2.810	1.930	4.790	2.790	2.780	2.810

Table S2: Z scores of all genes for a given time-course in EMT and non-EMT states of cells.

	- TGFβ						+ TGFβ					
TNFα→	Ctl	1hr	3Hr	6Hr	12Hr	24Hr	Ctl	1hr	3Hr	6Hr	12Hr	24Hr
NFKBa	-1.336	1.409	-0.618	-0.799	-0.947	-0.360	-1.098	1.639	0.170	-0.116	1.290	0.766
GroB	-0.695	-0.314	-0.599	-0.637	-0.634	-0.662	-0.564	3.010	0.317	-0.004	0.514	0.268
TNIP1	-1.234	-1.512	-0.364	0.620	0.014	-0.207	-1.312	-0.599	0.926	0.862	1.340	1.468
IL-6	-0.717	-0.654	-0.694	-0.688	-0.717	-0.717	-0.673	2.432	0.251	-0.096	0.970	1.303
IL-8	-0.540	-0.672	-0.867	-0.861	-0.854	-0.868	-0.807	1.998	0.714	0.293	1.269	1.196
TSLP	-0.740	3.049	0.518	-0.392	-0.263	-0.722	-0.579	-0.401	-0.219	-0.599	0.475	-0.128
TRAF1	-0.794	-0.749	-0.737	-0.740	-0.689	-0.336	-0.678	0.117	0.260	0.422	2.560	1.363
A20	-0.917	0.449	-0.346	-0.445	-0.778	-0.550	-0.895	2.889	-0.110	-0.138	0.736	0.105
TRAF3	-0.801	-0.961	-0.529	-0.674	-0.880	0.675	-0.119	-0.611	-0.505	0.757	2.373	1.274
INFB	-0.940	0.292	-1.028	-1.108	-0.060	-0.756	0.401	0.689	0.001	-0.803	2.494	0.817
IL-25	-0.035	-0.739	-0.703	-0.713	-0.735	0.570	0.196	-0.506	2.869	-0.246	0.043	NA
IL-33	0.669	-0.679	2.177	0.984	0.404	0.716	-0.910	-0.877	-0.654	NA	-0.837	-0.992
IFNa6	-1.001	0.158	-1.077	-1.241	-0.526	-0.782	0.869	0.736	0.124	-0.499	2.158	1.080
IFNa21	-0.958	0.492	-1.123	-1.122	-0.301	-0.662	0.707	0.556	0.131	-0.763	2.459	0.582
RANTES	-0.323	-0.526	-0.164	-0.103	3.253	0.032	-0.597	-0.510	-0.384	-0.297	0.001	-0.382
RIG-1	-0.242	-1.212	1.638	1.828	-0.104	1.307	-1.252	-0.825	-0.322	-0.405	0.047	-0.459
LMP2	-0.679	-1.221	-0.166	1.807	1.720	0.811	-1.211	-1.107	-0.380	0.326	0.362	-0.263
TAP1	-0.869	-1.257	0.386	1.207	0.386	1.577	-1.248	-1.210	-0.773	0.059	1.249	0.492
STAT1	0.566	-1.247	1.320	1.807	0.502	0.935	-0.947	-1.101	-1.141	-0.615	0.276	-0.355
STAT2	-0.719	-1.034	-0.842	-0.800	-0.703	-0.083	0.849	-0.177	-0.555	0.459	2.433	1.172
IRF1	-0.795	0.417	1.294	-0.020	-1.071	-0.685	-1.194	2.476	-0.324	-0.236	0.367	-0.228
IRF9	-0.414	-0.804	-0.594	0.231	-0.140	2.537	-0.383	-0.991	-1.110	-0.079	1.335	0.413
SOCS3	-0.953	1.193	-0.805	-0.923	-0.739	-0.822	0.458	0.405	-0.450	-0.626	2.204	1.059
Fos	-0.017	-0.194	-0.268	-0.352	-0.343	-0.287	-0.363	-0.382	-0.420	3.299	-0.313	-0.360
c-Jun	-0.982	-0.459	-1.079	-1.013	-0.848	-0.704	1.084	0.392	-0.017	0.257	2.105	1.263
SOD2	-0.943	0.276	-1.016	-0.996	1.677	0.787	0.260	-0.947	1.547	-0.733	0.960	-0.872
NOX1	-0.746	-0.423	-0.644	NA	1.004	-0.934	-0.611	-0.373	-0.504	-0.265	1.032	2.464
NOX4	-0.906	-0.907	-0.885	-0.907	-0.907	-0.907	1.900	1.104	0.671	-0.140	0.807	1.076
FSP1	2.395	-1.394	1.591	-0.720	-0.194	0.122	-0.552	0.156	-0.652	0.173	-0.520	-0.404
SMA	0.298	0.909	-0.761	-1.312	0.071	0.279	-0.122	-0.736	-1.605	-0.105	2.176	0.909
Col1A	-0.978	NA	NA	-0.984	-0.970	-0.921	0.875	NA	0.274	-0.066	1.934	0.836
Vimentin	-0.835	-0.905	-0.848	-0.848	-0.911	-0.849	1.138	0.368	0.262	0.164	2.198	1.066
Desmin	-0.265	-0.394	-0.338	-0.377	-0.303	-0.394	-0.275	-0.270	-0.336	3.157	NA	-0.204
MX1	-0.049	-0.709	0.638	2.228	0.758	1.512	-0.805	-0.821	-0.829	-0.699	-0.521	-0.704
IFI27	-1.350	0.300	-1.341	-1.332	1.485	0.831	0.497	0.110	0.348	-1.295	1.008	0.739
NFKB2	-1.476	-0.491	-0.718	0.240	0.328	-0.233	-1.380	-0.653	1.155	0.021	1.459	1.747
CDKN1A1	-1.092	-1.578	-1.092	-1.263	0.410	0.062	0.410	0.185	0.826	0.148	1.505	1.478
PCNA	-0.638	-1.611	-1.180	0.223	-1.087	-0.787	1.757	1.065	0.897	0.186	0.616	0.560
ITGA2	1.468	-1.528	1.068	-0.250	0.709	0.549	-1.368	-1.368	-0.689	-0.090	0.469	1.028
SRT39	0.942	2.437	-1.329	-0.498	-0.665	-0.332	-0.886	-0.554	-0.665	0.277	0.332	0.942
TM4SF1	-0.841	-1.252	-0.501	-0.125	-1.190	-0.841	-0.340	-0.081	0.662	1.494	1.494	1.521
ATM	-0.365	-1.323	-1.009	-0.899	-1.087	-0.852	1.535	1.299	0.294	0.341	1.095	0.969
IL-28A	-0.935	-0.551	-0.234	-0.359	-0.290	-0.765	-0.404	-0.284	-0.415	2.152	2.214	-0.129
IL-28B	-0.877	-0.336	0.426	-0.056	-0.426	-0.931	-0.581	-0.367	-0.783	1.822	2.331	-0.220
IL-29	-1.349	-0.259	1.271	0.690	-0.883	-1.311	-0.248	0.487	-0.796	2.027	0.640	-0.269
HOXB9	-0.758	1.085	-1.295	-1.537	-0.462	-0.220	0.153	0.756	-0.758	0.164	0.877	1.996
SLUG	-0.887	-0.952	-1.074	-0.675	-0.602	-0.887	0.601	0.666	0.276	-0.114	1.836	1.812
SNAL1	-0.921	-0.843	-0.729	-0.701	-0.877	-0.948	1.511	0.235	-0.064	0.095	1.890	1.352
TWIST1	-0.484	-0.727	-0.742	-0.724	-0.640	-0.687	0.146	2.900	0.484	-0.407	0.619	0.262
TWIST2	-0.493	-1.107	-0.849	-0.828	-1.016	-1.058	0.770	0.156	2.152	0.756	0.749	0.770
